# Dihydroartemisinin Exerts Antifibrotic and Anti-Inflammatory Effects in Graves’ Ophthalmopathy by Targeting Orbital Fibroblasts

**DOI:** 10.3389/fendo.2022.891922

**Published:** 2022-05-17

**Authors:** Shenglan Yang, Xing Wang, Wei Xiao, Zhihui Xu, Huijing Ye, Xiaotong Sha, Huasheng Yang

**Affiliations:** State Key Laboratory of Ophthalmology, Zhongshan Ophthalmic Center, Sun Yat-sen University, Guangdong Provincial Key Laboratory of Ophthalmology and Visual Science, Guangzhou, China

**Keywords:** dihydroartemisinin, Graves’ ophthalmopathy, orbital fibroblast, fibrosis, inflammation, STAT3, ERK

## Abstract

Graves’ ophthalmopathy (GO) is a common orbital disease that threatens visual function and appearance. Orbital fibroblasts (OFs) are considered key target and effector cells in GO. In addition, hyaluronan (HA) production, inflammation, and orbital fibrosis are intimately linked to the pathogenesis of GO. In this study, we explored the therapeutic effects of dihydroartemisinin (DHA), an antimalarial drug, on GO-derived, primary OFs. CCK8 and EdU assays were applied to evaluate the antiproliferative effect of DHA on OFs. Wound healing assays were conducted to assess OF migration capacity, while qRT-PCR, western blotting, ELISA, and immunofluorescence were used to determine the expression of fibrosis-related and pro-inflammatory markers in these cells. Moreover, RNA sequencing was conducted to identify differentially expressed genes (DEGs) in DHA-treated OFs, and Gene Ontology (GO) and Kyoto Encyclopedia of Genes and Genomes (KEGG) pathway enrichment analysis of DEGs was performed to explore potential mechanisms mediating the antifibrotic effect of DHA on GO-derived OFs. Results showed that DHA dose-dependently inhibited OF proliferation and downregulated, at the mRNA and protein levels, TGF-β1-induced expression of fibrosis markers, including alpha smooth muscle actin (α-SMA) and connective tissue growth factor (CTGF). Furthermore, DHA inhibited TGF-β1 induced phosphorylation of extracellular signal-regulated protein kinase 1/2 (ERK1/2) and signal transducer and activator of transcription 3 (STAT3), which suggested that DHA exerted antifibrotic effects *via* suppression of the ERK and STAT3 signaling pathways. In addition, DHA suppressed the expression of pro-inflammatory cytokines and chemokines, including IL-6, IL-8, CXCL-1, MCP-1, and ICAM-1, and attenuated HA production induced by IL-1β in GO-derived OFs. In conclusion, our study provides first-time evidence that DHA may significantly alleviate pathogenic manifestations of GO by inhibiting proliferation, fibrosis- and inflammation-related gene expression, and HA production in OFs. These data suggest that DHA may be a promising candidate drug for treatment of GO.

## 1 Introduction

Graves’ ophthalmopathy (GO) is the most common and serious extrathyroidal manifestation of Graves’ disease, an autoimmune disorder (1). GO patients often go through symptoms such as dry eye, photophobia, excessive tearing, double vision, and a pressure sensation behind the eyes. The main clinical manifestations of GO are upper eyelid retraction, edema, erythema of the periorbital tissues and conjunctiva, and proptosis (2). Approximately 3% to 5% of GO patients have severe disease including sight-threatening corneal ulceration or compressive optic neuropathy (3). This not only dramatically reduces the life quality of patients, but also causes labor loss and entails a social and economic load.

The pathogenesis of GO remains unclear. Previous studies showed that many cell types, such as B cells, T cells, macrophages, mast cells, and orbital fibroblasts (OFs), are involved in the development and progression of GO (4–7). Among those, OFs are considered as both target and effector cells (4, 8). Compared to OFs from healthy individuals, GO-associated OFs overexpress thyroid-stimulating hormone receptor (TSHR) and insulin-like growth factor-1 receptor (IGF-1R), which are considered important autoimmune targets (9–11). Once OFs are activated, they release chemokines and cytokines, including IL-1β, IL-2, IL-6, CXCL8, IL-10, IL-17A, COX2, CCL2, CCL5, IFN-γ, TNF-α, and TGF-β (12, 13), which act on immunocytes to aggravate the inflammatory response. These interactions ultimately result in OFs expressing extracellular matrix molecules and proliferating and differentiating into myofibroblasts or lipofibroblasts, leading to tissue fibrosis and orbital remodeling (14).

Glucocorticoids (GCs) are currently the first-line therapy for moderate-to-severe active GO (15). However, GCs can cause dose-limiting adverse reactions such as weight gain, hypertension, cushingoid features, osteonecrosis, liver damage, and cardiovascular complications (16–19). In addition, some patients are GC-refractory or develop GC dependency (20). Biological agents targeting immunocytes and/or cytokines, such as rituximab (RTX, a monoclonal antibody to the antigen CD20) (21–23), etanercept (an inhibitor of TNF-α) (24), and adalimumab (an anti-TNF antibody) (25) are also used for active GO treatment. However, only a few small clinical retrospective studies have been conducted, and additional randomized controlled trials (RCTs) are needed to determine the efficacy and safety profile of these drugs. Recently, novel drugs targeting key surface receptors in OFs (such as TSHR and IGF-1R) have emerged. Teprotumumab, an IGF-1R-inhibiting monoclonal antibody, was associated with better outcomes than placebo with respect to proptosis, clinical activity score (CAS), diplopia, and quality of life in a phase 3 multicenter RCT (26). Nonetheless, teprotumumab use also has limitations, such as high cost and unproved efficacy on inactive GO. Limitations of current treatment modalities for GO extend not only to orbital radiotherapy and novel immunosuppressive therapies, as orbital decompression surgery, conventionally used to alleviate symptoms in the inactive phase of GO, is also often associated with complications. Therefore, more effective treatments for GO need to be developed.

This study investigated the therapeutic effects of dihydroartemisinin (DHA) in an *in vitro* model of GO. DHA is a derivative of artemisinin, a sesquiterpene lactone widely used for the treatment of fever and malaria that is extracted from *Artemisia annua L*, a traditional Chinese herb (27, 28). Clinical use of DHA as an antimalarial drug proved to be safe and tolerable, with good absorption, wide distribution, and rapid metabolism. Recently, DHA has been found to have other therapeutic actions besides its antimalarial effects. A study has shown that DHA improved histopathological features of liver fibrosis in rats by decreasing collagen deposition, inducing the senescence of activated hepatic stellate cells, and reducing the fibrogenic response to damage (29). Based on its ability to suppress inflammatory chemokine production, reduce inflammatory cell infiltration, and relieve disease activity in lupus nephritis, it was also suggested that DHA may be useful in the treatment of systemic lupus erythematosus (30, 31). Furthermore, DHA was shown to attenuate lipopolysaccharide-induced acute kidney injury *via* inhibition of inflammatory mediators and oxidative stress (32).

Based on the above evidence, we hypothesized that DHA might alleviate GO symptoms by eliciting antifibrotic and anti-inflammatory effects on OFs. Through cellular, molecular, and computational analyses on primary OF cultures from GO and non-GO patients, the present study provides robust evidence for the therapeutic potential of DHA as a treatment for GO.

## 2 Materials And Methods

### 2.1 Primary Cell Culture and Treatments

Orbital connective tissue samples were collected from six GO patients who underwent orbital decompression and from six patients without GO who underwent eyeball enucleation. The baseline characteristics of all patients are listed in **Table 1**. The severity and clinical activity of GO were graded according to the NOSPECS classification and the 7-item clinical activity score (CAS) scheme proposed by EUGOGO, respectively (15, 33). All patients signed informed consent forms. This study was conducted in accordance with the Declaration of Helsinki and was approved by the Institutional Review Board of Zhongshan Ophthalmic Center (2016KYPJ028).

**Table 1:** Clinical features of patients samples included in this study.

Age (years)	Sex (M/F)	CAS	GO severity assessment		Duration of GO (months)	Proptosis (R/L, mm)	Previous treatment	Surgery performed
GO patients								
46	M	2	IV	8		21/20	GCs	Decompression
53	F	1	IV	24		17/16	GCs	Decompression
33	F	0	III	19		20/21	GCs	Decompression
65	M	5	VI	12		24/24	GCs	Decompression
42	F	0	VI	26		23/22	GCs	Decompression
39	M	3	III	6		24/21	GCs	Decompression
Non-GO patients								
56	F	–	–	–		–	–	Enucleation
67	F	–	–	–		–	–	Enucleation
49	M	–	–	–		–	–	Enucleation
55	F	–	–	–		–	–	Enucleation
57	M	–	–	–		–	–	Enucleation
48	M	–	–	–		–	–	Enucleation

CAS, clinical activity score; M/F, male or female; GO, Graves’ ophthalmopathy; R/L, right or left eyes; GCs, glucocorticoids.

Orbital connective tissues obtained from surgery were cut into small pieces and plated in 10-cm culture dishes in high glucose Dulbecco’s Modified Eagle’s Medium (DMEM) containing 20% fetal bovine serum (FBS) and 1% penicillin/streptomycin (all from Gibco Laboratories, New York, USA) at 37°C in a 5% CO_2_ humidified incubator. After the cells had migrated out of the tissue pieces and reached confluence, they were passaged with 0.25% trypsin/EDTA (Gibco Laboratories, New York, USA). The cultured cell strains were used between the third and sixth passages. We replaced the culture medium with medium containing 1% FBS before stimulation with TGF-β1 (10 ng/mL) (R&D Systems, Minneapolis, MN, USA) or IL-1β (1ng/mL) (Cell Signaling Technology, Boston, MA, USA). In some experiments, cells were pretreated with DHA (Selleck Chemicals, Shanghai, China), U0126 (ERK inhibitor) (Cell Signaling Technology, Boston, MA, USA), and Stattic (STAT3 inhibitor) (MedChemExpress, Shanghai, China). Detailed experimental conditions are outlined in the main text or figure legends. Each experiment was repeated on at least three independent specimens.

### 2.2 Cell Proliferation Assays

Cellular proliferation was assessed using a Cell Counting Kit-8 (CCK-8) assay kit (Bimake Biotechnology, Houston, Texas, USA). Cells were seeded in 96-well plates and treated with different concentrations of DHA for the indicated times. CCK-8 reagent diluted at a 10:1 ratio was then added to the medium, and the cells were cultured for another 2 h before measuring well absorbance at 450 nm using a Synergy H1 microplate reader (Berten instrument company, Vermont, USA).

DNA synthesis was determined by the 5-ethynyl-2′-deoxyuridine (EdU) incorporation assay using a BeyoClick™ EdU Cell Proliferation Kit with Alexa Fluor 488 (Beyotime Biotechnology, Shanghai, China). Briefly, cells were seeded in 24-well plates and treated with different concentrations of DHA for 24 h. Cells were then exposed to EdU for 2 h, fixed in 4% paraformaldehyde, permeabilized in 0.3% Triton X-100, and stained according to the manufacturer’s instructions. Then counterstained with 4′,6-diamidino-2-phenylindole (DAPI) (Servicebio, Wuhan, Hubei, China) for 5 min. Images were taken using a fluorescence microscope (Nikon Ts2FL, Tokyo, Japan) at 488 nm excitation.

### 2.3 RNA Extraction and Quantitative Reverse Transcriptase-Polymerase Chain Reaction (qRT-PCR)

Total RNA was extracted from OFs using an RNA Quick Purification kit (ESscience Biotechnology, Shanghai, China). RNA was reverse-transcribed into cDNA using a PrimeScript RT Reagent Kit (TaKaRa, Dalian, Liaoning, China). Quantitative reverse transcriptase-polymerase chain reaction (qRT-PCR) was performed using a SYBR Fast qPCR kit (Kapa Biosystems, Boston, MA, USA) according to the manufacturer’s instructions. Glyceraldehyde phosphate dehydrogenase (GAPDH) was used as internal control. The primer pair sequences for qRT-PCR are listed in **Table 2**. The data are presented as the means ± SD of triplicate values from three independent experiments.

**Table 2:** Primer sequences of qRT-PCR.

Genes	Sequences (5’-3’)
GAPDH	F:TTGCCATCAATGACCCCTT R:CGCCCCACTTGATTTTGGA
α-SMA(ACTA2)	F:GAA CCC TAA GGC CAA CCG GGA GAA A R:CCA CAT ACA TGG CGG GGA CAT TGA
CTGF	F:AGC TGA CCT GGA AGA GAA CAT T R:GCT CGG TAT GTC TTC ATG CTG
COL1A1	F:AAAGATGGACTCAACGGTCTC R:CATCGTGAGCCTTCTCTTGAG
FN1	F:ACAAGCATGTCTCTCTGCCAA R:GCAATGTGCAGCCCTCATTT
IL-6	F:CACTGGTCTTTTGGAGTTTGAG R:GGACTTTTGTACTCATCTGCAC
IL-8	F:CCACCGGAAGGAACCATCTC R:GGGGTGGAAAGGTTTGGAGT
CXCL1	F:TTCACAGTGTGTGGTCAACAT R:AAGCCCCTTTGTTCTAAGCCA
MCP-1	F:CCTTCATTCCCCAAGGGCTC R:CTTCTTTGGGACACTTGCTGC
ICAM-1	F:TGCAAGAAGATAGCCAACCAAT R:GTACACGGTGAGGAAGGTTTTA
HAS1	F:GCGGGCTTGTCAGAGCTAC R:ACTGCTGCAAGAGGTTATTCC
HAS2	F:CCTCCTGGGTGGTGTGATTT R:GCGTCAAAAGCATGACCCAA
HAS3	F:TTATACAGCTTTTCTACCGGGG R:CAGAAGGCTGGACATATAGAGG

F, forward; R, reverse.

### 2.4 Western Blotting

A protein extraction kit (KeyGEN Biotech, Nanjing, Jiangsu, China) was used to lyse cultured OFs. Protein concentrations were measured with a BCA kit (Beyotime Biotechnology co., Shanghai, China). Equal amounts of protein samples were separated by sodium dodecyl sulfate–polyacrylamide gel electrophoresis (SDS-PAGE), transferred to a polyvinylidene fluoride (PVDF) membrane, and incubated overnight at 4°C with the following primary antibodies: GAPDH (D16H11) XP^®^ Rabbit mAb, β-Tubulin (9F3) Rabbit mAb, anti-alpha smooth muscle actin antibody [1A4] (α-SMA), CTGF (D8Z8U) Rabbit mAb, Smad2 (D43B4) XP^®^ Rabbit mAb, phospho-Smad2 (Ser465/467) (138D4) Rabbit mAb, Smad3 (C67H9) Rabbit mAb, phospho-Smad3 (Ser423/425) (C25A9) Rabbit mAb, Stat3 (124H6) Mouse mAb, Phospho-Stat3 (Tyr705) (D3A7) XP^®^ Rabbit mAb, p44/42 MAPK (Erk1/2) (137F5) Rabbit mAb, Phospho-p44/42 MAPK (Erk1/2) (Thr202/Tyr204) (D13.14.4E) XP^®^ Rabbit mAb. All antibodies were purchased from Cell Signaling Technology (Boston, MA, USA), except for anti-α-SMA antibody, which was from Abcam (Cambridge, UK). HRP-conjugated goat anti-rabbit and horse anti-mouse secondary antibodies (Cell Signaling Technology, Boston, MA, USA) were used for detection. Blots were visualized using enhanced chemiluminescence (WBKLS0500, Merck Millipore, MA, USA). PageRuler™ Prestained Protein Ladders #26616, #26619, and #26625 from ThermoFisher Scientific (Darmstadt, Germany) were used to determine molecular weights. Bands were quantified using ImageJ Software (NIH, Bethesda, MA, USA).

### 2.5 Immunofluorescence

OFs were seeded on coverslips within 6-well plates (approximately 5 ×10^4^ cells per well). After the cells reached 50% to 70% confluence, they were treated with DHA (20 μM) with or without TGF-β1 (10 ng/mL). Unstimulated cells served as control. Following treatment for 48h, cells were washed twice with phosphate buffered saline (PBS), fixed with 4% paraformaldehyde for 20 min, permeabilized with 0.3% Triton X-100, blocked with 3% BSA for 30 min at room temperature, and incubated with primary antibodies against COL1A1 (E6A8E) Rabbit mAb (Cell Signaling Technology, Boston, MA, USA) and anti-α-SMA (Abcam, Cambridge, UK). Cells were then washed, incubated with the Alexa Fluor 488 or Alexa Fluor 546-labeled secondary antibody (both from Nanoprobes, New York, USA) for 1 h in the dark at room temperature, and counterstained with DAPI (Servicebio, Wuhan, Hubei, China) for 5 min. Finally, anti-fading agent was added to mount the coverslips. Confocal microscopy (Carl Zeiss 880, Oberkochen, Germany) was used to take immunofluorescence images.

### 2.6 Wound Healing Assay

OFs (1 × 10^5^ cells per well) were seeded into 6-well plates and grown to confluence. A straight-line scratch was created using a sterile 200 μL disposable micropipette tip. Cellular debris was removed by washing with PBS twice, and cultures were incubated in DMEM containing 1% FBS. The cells were then treated with DHA (0, 20 μM) with or without TGF-β1 (10 ng/mL) and defined microscopic scratch wound areas were photographed at the indicated times using an inverted phase-contrast microscope.

### 2.7 Hyaluronan (HA) Measurement

GO-derived OFs were treated with different concentrations of DHA (0, 10, 20 μM) and 1 ng/mL IL-1β (R&D Systems, Minneapolis, MN, USA) in DMEM supplemented with 1% FBS. After 24 h, cell culture supernatants were collected and centrifuged at 5,000 g for 10 min. HA concentrations in cell culture supernatants were detected with an ELISA kit (R&D Systems, Minneapolis, MN, USA) following the manufacturer’s instructions.

### 2.8 RNA Sequencing

#### 2.8.1 RNA Isolation and Library Preparation

OFs were pretreated with DHA (0, 20 μM) for 3 h and then stimulated with or without TGF-β1 (10 ng/mL) (n=3 cultures/treatment) for 48 h. Total RNA was extracted using a mirVana miRNA Isolation Kit (Ambion) according to the manufacturer’s protocol. RNA purity and quantification were evaluated using a NanoDrop 2000 spectrophotometer (Thermo Scientific, USA). RNA integrity was assessed using an Agilent 2100 Bioanalyzer (Agilent Technologies, Santa Clara, CA, USA). RNA-Seq libraries were constructed using TruSeq Stranded mRNA LT Sample Prep Kit (Illumina, San Diego, CA, USA) according to the manufacturer’s instructions. Transcriptome sequencing and analysis were conducted by OE Biotech Co., Ltd. (Shanghai, China).

#### 2.8.2 RNA Sequencing and Analysis of Differentially Expressed Genes (DEGs)

The cDNA libraries were sequenced on an Illumina HiSeq X Ten platform and 150 bp paired-end reads were generated. About 47,029,247 total raw reads for each sample were obtained. Raw data (raw reads) in fastq format were first processed using Trimmomatic (34) to remove low quality reads, and approximately 46,486,966 clean reads for each sample were retained for subsequent analyses. The clean reads were mapped to the human genome (GRCh38; https://www.ncbi.nlm.nih.gov/assembly/GCF_000001405.26/) using HISAT2 (35). FPKM values (36) of each gene were calculated using Cufflinks (37) and corresponding read counts were obtained by HTSeq-count (38). Differential expression analysis was performed using the DESeq (2012) R package. P < 0.05 and fold change > 1.5 and < 0.67 were set as thresholds for significantly differential expression. Hierarchical cluster analysis of differentially expressed genes (DEGs) was performed to demonstrate the expression pattern of genes in different groups and samples. Gene ontology (GO) enrichment and Kyoto Encyclopedia of Genes and Genomes (KEGG) (39) pathway enrichment analysis of DEGs were performed based on hypergeometric distribution using R. Enrichment score > 1.0 and p < 0.05 were set for the identification of GO terms. P < 0.05 was set as the cut-off value on KEGG analysis. Among the top 50 enriched GO terms and KEGG pathways, 10 or 15 terms were selected and drawn using the ggplot2 R package.

#### 2.8.3 TRRUST Analysis

Core regulatory transcription factors (TFs) influenced by DHA were predicted based on the RNA-seq data. The web-based portal Metascape (https://www.metascape.org) was used to conduct TRRUST analysis, incorporating downregulated DEGs as input parameters (40). The top 20 predicted TFs regulating the DHA-downregulated DEGs were drawn using the ggplot2 R package.

### 2.9 Statistical Analysis

Each experiment was performed in triplicate, and data are expressed as the means ± SD. GraphPad Prism software was used to analyze the results. Statistical analyses were performed by one-way ANOVA or two-way ANOVA, followed by Tukey’s multiple comparison test or Sidak’s multiple comparisons test. P < 0.05 indicated a statistically significant difference.

## 3 Results

### 3.1 OF Isolation and Identification

Primary OF cultures were established from orbital connective tissues resected from GO and non-GO patients (**Table 1**). Vimentin, which is present in cells of mesoderm origin such as fibroblasts, was primarily used to identify OFs (**Supplementary Figure 1**). In addition, negative expression of S100, desmin, and cytokeratin served to confirm the exclusion of nerve cells, skin melanocytes, smooth muscle cells, cardiomyocytes, and epithelial cells in these cultures (**Supplementary Figure 1**).

### 3.2 DHA Inhibits OF Proliferation

**Figure 1:**
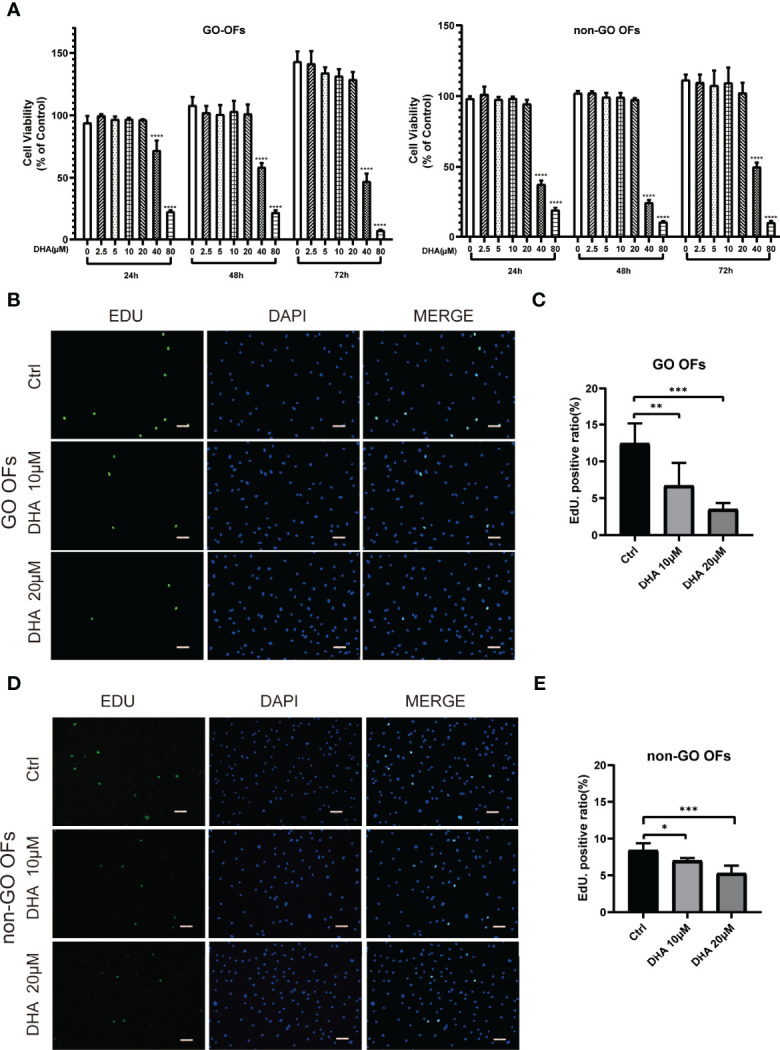
Effect of DHA on the cellular viability of OFs from GO and non-GO patients. **(A)** OFs obtained from 3 GO patients and 3 non-GO patients were treated with increasing concentrations of DHA (0, 2.5, 5, 10, 20, 40, and 80 μM) for 24, 48, and 72 h, respectively. Cell viability is presented as the percentage relative to the viability of the untreated cells. **(B, D)** Representative images of the EdU assay results in GO OFs and non-GO OFs treated with different concentrations of DHA (10, 20 μM). The cells were observed using a fluorescence microscope, scale bars = 50 μm. Green: EdU, Blue: 4′,6-diamidino-2-phenylindole (DAPI). **(C, E)** Quantification of the EdU assay results of GO OFs and non-GO OFs(Ctrl, DHA [10, 20 μM], n = 5). For **(A, C)**, and **(E)**, the summarized data are reported as the mean ± standard error of the mean. *P < 0.05, **P < 0.01, ***P < 0.001, **** P < 0.0001, versus the Ctrl group.

The effect of DHA on the proliferation of OFs was evaluated using both CCK-8 and EdU assays. CCK-8 assay results showed that at concentrations ≥ 40 μM, DHA significantly inhibited the proliferation of both GO OFs and non-GO OFs at 24, 48, and 72 h post-treatment (**Figure 1A**). Since DHA ≤ 20 μM did not affect the viability of GO and non-GO OFs, 10 and 20 μM DHA doses were applied in subsequent experiments. EdU assays showed that the basal OF proliferation rate was slightly higher in GO than in non-GO OF cultures (P < 0.01). Additionally, results showed that DHA suppressed proliferation and DNA synthesis in both GO and non-GO OFs in a dose dependent manner (**Figures 1B** **–E**). Moreover, we calculated the decrease in the proportion of EdU positive cells after DHA treatment in both GO OFs and non-GO OFs. Results indicated that the inhibitory effect of DHA on cell proliferation was significantly stronger in GO-OF compared with non-GO OFs (P < 0.05) (**Supplementary Figure 2**).

### 3.3 DHA Exerts Antifibrotic Effects on OFs

**Figure 2:**
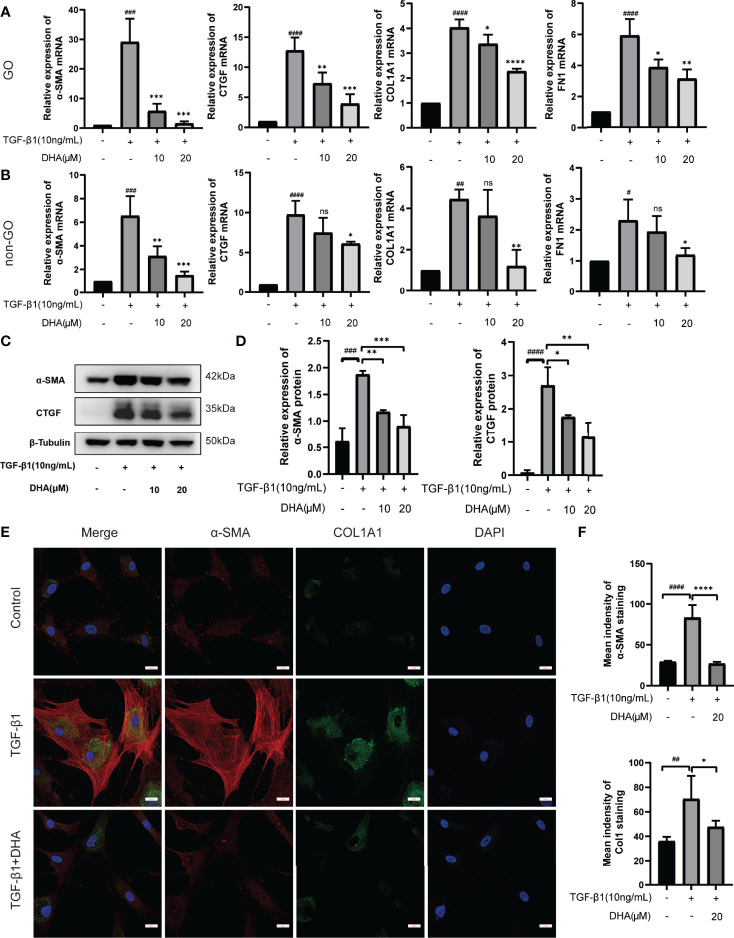
DHA exerts antifibrotic effects on OFs. **(A, B)** The mRNA levels of α-SMA, CTGF, COL1A1, and FN1 in GO and non-GO OFs (n = 3). **(C)** The protein expression levels of α-SMA and CTGF were determined using western blot analysis (n = 3). **(D)** The densities of α-SMA and CTGF protein bands were quantified and normalized to β-Tubulin. **(E)** Representative images of α-SMA and COL1A1 immunostaining in GO OFs treated with TGF-β1 (10 ng/mL, 48 h) with or without pretreatment of DHA (20 μM, 3 h). The cells were observed using a confocal microscope. Scale bars = 25 μm. Red: α-SMA, Green: COL1A1, Blue: DAPI. **(F)** Quantification of mean idensity of α-SMA and COL1A1. For **(A, B, D, F)**, the summarized data are reported as the mean ± standard error of the mean. ^#^P < 0.05, ^# #^P < 0.01, ^# # #^P < 0.001, ^# # # #^P <0.0001 compared with the control; *P < 0.05, **P < 0.01, ***P < 0.001, ****P < 0.0001 compared with TGF-β1 alone. ns denotes no statistical significance versus the control/TGF-β1.

To determine whether DHA exerts antifibrotic effects on OFs, the expression of fibrosis-related genes was next examined. As shown in **Figures 2A, B**, following stimulation with TGF-β1 (10 ng/mL) for 48 h, α-SMA, CTGF, COL1A1, and FN1 mRNA levels were markedly increased in both GO OFs and non-GO OFs. Notably, the mRNA expression of α-SMA and FN1 were significantly higher in GO TGF-β1-treated groups than in non-GO TGF-β1-treated groups (P < 0.0001). However, pre-treatment of cultures with DHA (10 or 20 μM) for 3 h resulted in significant, dose-dependent downregulation of these markers. Moreover, western blot analysis revealed that DHA exposure suppressed α-SMA and CTGF expression at the protein level in OFs subjected to TGF-β1 stimulation (**Figures 2C, D** **)**. The antifibrotic effects of DHA were further assessed by double immunofluorescence staining of α-SMA and COL1A1. As shown in **Figures 2E, F**, TGF-β1-mediated upregulation of both α-SMA (localized to the cytoskeleton) and COL1A1 (distributed in the cytoplasm) was remarkably repressed by DHA exposure.

### 3.4 DHA Suppresses the Migration Capacity of GO-Derived OFs

**Figure 3:**
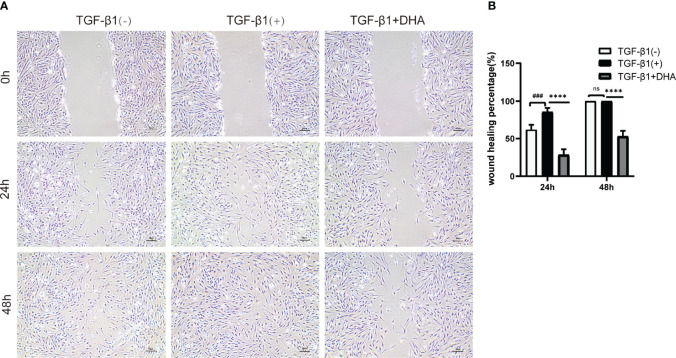
DHA suppresses the migration capacity of GO-derived OFs. **(A)** Representative images of wound repair ability of GO OFs (n = 3). Scale bar = 50 μm. **(B)** Statistical analysis of the rate of wound closure. ^# # #^P < 0.001 compared with the control; *P < 0.05, ****P < 0.0001 compared with TGF-β1 alone. ns denotes no statistical significance versus the TGF-β1.

Wound healing assays were next performed to assess the effect of DHA on the migration ability of GO-derived OFs. As shown in **Figure 3**, by 24 h post-treatment wound closure was slightly higher in TGF-β1-treated compared to control OFs. After 48 h, the wounds were nearly closed in both control and TGF-β1 cultures. In contrast, at both time points, wound closure was significantly delayed in DHA-treated OFs compared with non-DHA-treated cells.

### 3.5 DHA Exerts an Anti-Fibrotic Effect on GO-Derived OFs by Repressing Activation of the MAPK/ERK and STAT3 Signaling Pathways

**Figure 4:**
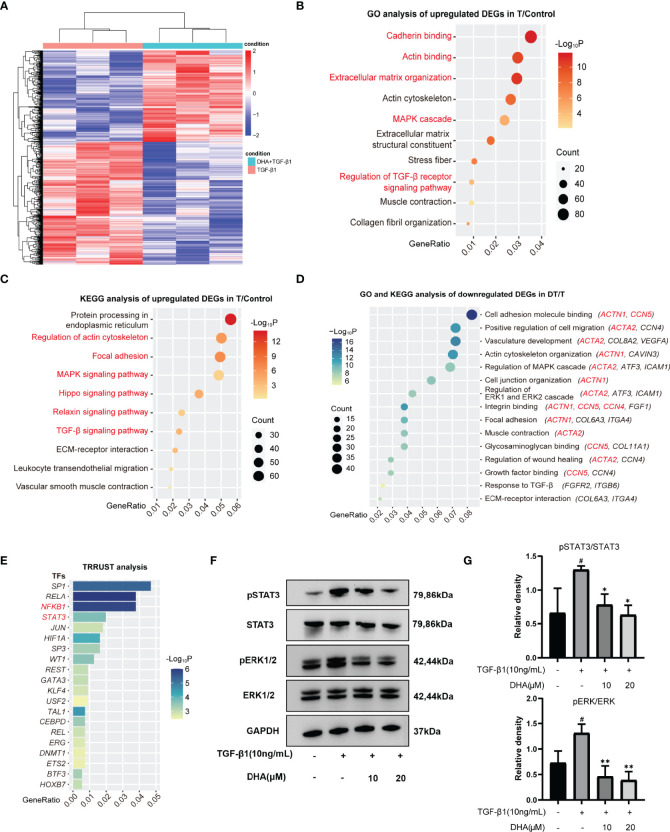
DHA suppresses TGF-β1-induced phosphorylation levels of ERK1/2 and STAT3. **(A)** Differentially expressed genes (DEGs) were explored and presented in heatmap of mRNA abundance. Columns denoting GO OFs grouped by TGF-β1(10 ng/mL, 48 h) stimulation with or without a 3-h pretreatment of DHA (20 μM) were sorted by diversity within the TGF-β1 group (n=3) and the TGF-β1+DHA group (n=3); Rows denoting RNA expression according to their enrichment in TGF-β1 versus TGF-β1+DHA. **(B, C)** Bubble chart from GO analysis or KEGG analysis showed TGF-β1-stimulated genes were mainly associated with cell differentiation, migration, and extracellular matrix organization. **(D)** Bubble chart from GO-KEGG analysis showed DHA significantly downregulates fibrosis-associated processes. The MAPK signaling and ERK1/2 signaling were decreased in TGF-β1+DHA group. **(E)** The top 20 transcription factors differentially regulated by DHA treatment were analyzed by TRRUST. **(F)** The protein levels of p-ERK, ERK, p-STAT3, STAT3, GAPDH in GO OFs stimulated with TGF-β1 (10 ng/mL, 3 h) with or without a 3-h pretreatment with DHA (10, 20 μM) were determined using western blot analysis (n = 3). **(G)** The protein levels of p-ERK and p-STAT3 were quantified and analyzed. ^#^P < 0.05 compared with the control; *P < 0.05, **P < 0.01 compared with TGF-β1 alone.

To explore the molecular mechanism involved in the antifibrotic effect elicited by DHA on GO-derived OFs, RNA sequencing was first performed to identify DEGs. A total of 2589 DEGs were detected between cells treated with TGF-β1 alone and those pre-treated with DHA (**Figure 4A**). We further performed function enrichment analyses of these DEGs using the KEGG and GO databases. As shown in **Figures 4B, C**, TGF-β1-stimulated genes were mainly associated with cell differentiation, migration, and extracellular matrix organization. In turn, pre-treatment with DHA correlated with significant downregulation of genes associated with the MAPK signaling pathway, the ERK1 and ERK2 signaling pathways, as well as fibrosis-associated processes, including OF activation, differentiation, migration, and extracellular matrix organization (**Figure 4D**). Based on TRRUST analysis, the top 20 transcription factors differentially regulated by DHA treatment are shown in **Figure 4E**.

Next, western blotting was performed to further investigate whether the MAPK/ERK signaling pathway is involved in the antifibrotic effects of DHA on GO-derived OFs. As shown in **Figures 4F, G**, DHA treatment inhibited TGF-β1-induced phosphorylation of ERK1/2. Previous studies showed that TGF-β1 can promote fibroblast activation and tissue fibrosis by both canonical Smad and non-Smad pathways (41) and that STAT3 activation also plays an important role in TGF-β1 signaling (42). Therefore, we also examined the phosphorylation of Smad2, Smad3, and STAT3 by western blotting. Results demonstrated that DHA obviously reduced TGF-β1-induced phosphorylation of STAT3 (**Figures 4F, G** **)**; however, DHA treatment had a non-significant effect on the phosphorylation of Smad2 and Smad3 (Data not shown).

**Figure 5:**
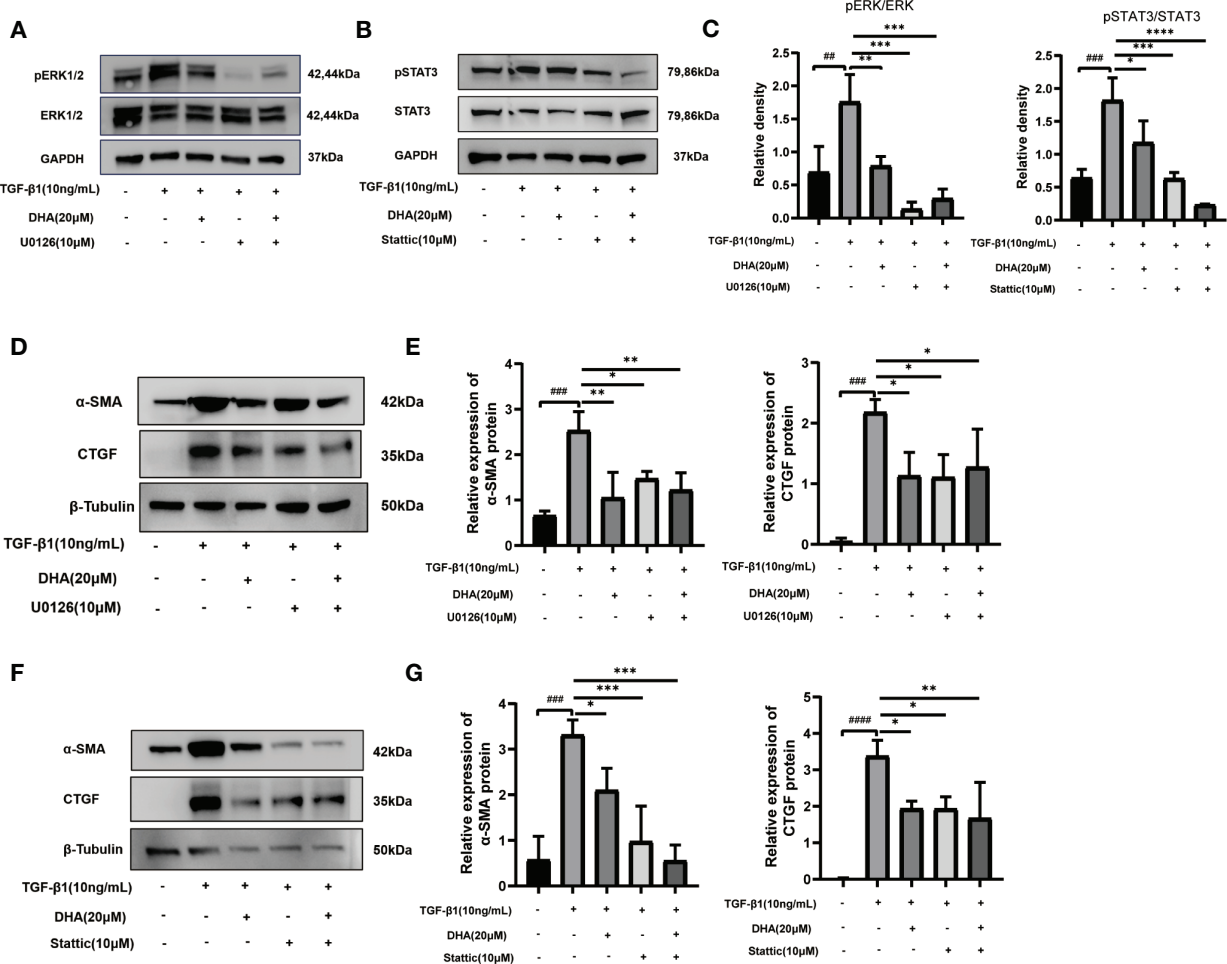
DHA exerts an antifibrotic effect on GO-derived OFs by repressing activation of the MAPK/ERK and STAT3 signaling pathways. **(A, B)** GO-derived OFs were stimulated with TGF-β1 (10 ng/mL, 3 h) with or without a 3-h pretreatment with DHA (20 μM) or ERK inhibitor U0126 (10 μM) or STAT3 inhibitor Stattic (10 μM). The expression and phosphorylation levels of ERK1/2 and STAT3 were determined using western blot analysis (n = 3). **(C)** The protein levels of pERK1/2 and pSTAT3 were quantified and analyzed. **(D)** GO-derived OFs were stimulated with TGF-β1 (10 ng/mL, 48 h) with or without a 3-h pretreatment with DHA (20 μM) or ERK inhibitor U0126 (10 μM). The expression levels of α-SMA and CTGF were determined using western blot analysis (n = 3). **(E)** The protein levels of α-SMA and CTGF were quantified and analyzed. **(F)** GO-derived OFs were stimulated with TGF-β1 (10 ng/mL,48 h) with or without a 3-h pretreatment with DHA (20 μM) or STAT3 inhibitor Stattic (10 μM). The expression levels of α-SMA and CTGF were determined using western blot analysis (n = 3). **(G)** The protein levels of α-SMA and CTGF were quantified and analyzed. For **(C, E, G)**, the summarized data are reported as the mean ± standard error of the mean. ^# #^P < 0.01, ^# # #^P < 0.001, ^# # # #^P < 0.0001 compared with the control; *P < 0.05, **P < 0.01, ***P < 0.001, ****P < 0.0001 compared with TGF-β1 alone.

To confirm whether ERK and STAT3 inactivation are responsible for the antifibrotic effect of DHA on GO-derived OFs, the ERK inhibitor U0126 and the STAT3 inhibitor Stattic were applied to cultured cells. As shown in **Figures 5A–C**, U0126 and Stattic effectively inhibited the phosphorylation of ERK1/2 and STAT3, respectively. Additionally, both inhibitors suppressed TGF-β1-induced α-SMA and CTGF protein expression (**Figures 5D–G**). Notably, DHA exposure mimicked the effects of U0126 and Stattic by blocking TGF-β1-induced ERK and STAT3 signaling in GO-derived OFs. These data indicate that DHA exerts antifibrotic effects on GO-derived OFs through inhibiting TGF-β1-induced MAPK/ERK and STAT3 signaling.

### 3.6 DHA Inhibits IL-1β-Induced Inflammation and Attenuates HA Production by OFs

**Figure 6:**
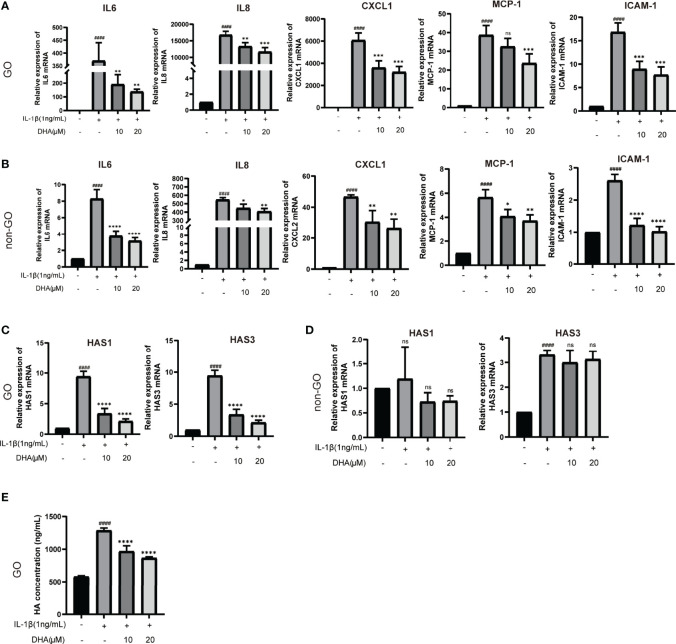
DHA inhibits IL-1β-Induced inflammation and attenuates HA production by OFs. GO OFs and non-GO OFs were stimulated with IL-1β (1 ng/mL,24 h) with or without a 3-h pretreatment with DHA (10, 20 μM). **(A, B)** The mRNA levels of IL-6, IL-8, CXCL1, MCP-1, and ICAM-1 were detected by qRT-PCR (n = 3). **(C, D)** The mRNA expression levels of HAS1 and HAS3 were detected by qRT-PCR (n = 3). **(E)** The concentrations of HA in the GO-derived cell culture supernatants were quantified by ELISA (n = 4). For **(A–E)**, the summarized data are reported as the mean ± standard error of the mean. ^# # # #^P < 0.0001 compared with the control; *P < 0.05, **P < 0.01, ***P < 0.001, ****P < 0.0001 compared with IL-1β alone. ns denotes no statistical significance versus the control/TGF-β1.

To investigate whether DHA also exerts anti-inflammatory effects on OFs, we applied qRT-PCR to detect the expression of inflammation-related genes. The changes observed in GO-derived OFs were more remarkable than those seen in non-GO OFs after stimulated by IL-1β (P < 0.0001). Results showed that DHA significantly suppressed IL-1β-induced mRNA expression of inflammatory markers, including IL-6, IL-8, CXCL-1, MCP-1, and ICAM-1, in OFs from both GO and non-GO patients (**Figures 6A, B** **)**.

Hyaluronic acid (HA) is a hydrophilic glycosaminoglycan importantly involved in GO pathogenesis by promoting the enlargement of extraocular muscle bodies (43, 44). Therefore, we detected the mRNA expression of core genes associated with HA production, namely HAS1, HAS2, and HAS3, in cultured OFs. As shown in **Figure 6C**, HAS1 and HAS3 mRNA expression was upregulated in IL-1β-stimulated, GO-derived OFs, and this effect was inhibited by DHA pre-treatment. In contrast, we found no significant changes in the expression of these genes in non-GO OFs (**Figure 6D**). Furthermore, we detected HA production in culture supernatants from GO-derived OFs *via* ELISA. Results showed that IL-1β increased HA production in GO-derived OFs, whereas DHA pre-treatment decreased this effect in a dose-dependent manner (**Figure 6E**). These data demonstrated that DHA inhibits IL-1β-induced inflammation and attenuates HA production by GO-derived OFs.

## 4 Discussion

GO is a visual threatening eye disease that may seriously affect the appearance and quality life of those affected. The main pathological features of GO are proliferation of orbital adipocytes and myofibroblasts, increased secretion of extracellular matrix components (especially HA), and autoimmune tissue damage mediated by infiltration of inflammatory cells. Although novel therapies for GO treatment have been proposed, its treatment remains challenging. Many clinical signs and symptoms of GO arise from soft-tissue enlargement in the ocular orbit, leading to increased pressure within the bony cavity (2, 44, 45). Orbital tissue enlargement in GO is determined by several factors, including rapid proliferation and excessive glycosaminoglycan (especially HA) production by OFs, and tissue remodeling and fibrosis during the late disease phase (46, 47). In our study, we provide evidence for the antifibrotic effects of DHA on GO *in vitro* and investigate the underlying mechanisms. Moreover, our data demonstrate that DHA remarkably attenuates IL-1β-induced HA production and secretion of pro-inflammatory mediators by GO-derived OFs.

Because of the high cost and time involved in developing new drugs, the repurposing of old drugs to expand their therapeutic applications is of great clinical interest. The safety of DHA, a major active metabolite of artemisinin, has been reassured by its widespread use in malaria patients. Clinical trials showed that after treatment with artemisinin and its derivatives, blood peak concentrations of DHA range between 2.2 and 21.3 μM (48–51). In our *in vitro* experiments, we mostly used two DHA concentrations (10 and 20 μM) matching DHA therapeutic physiological concentrations, which further underlines the potential value of DHA for GO treatment. GO patients are classified as type I and type II based on predominant enlargement of the orbital fat compartment or extraocular muscles, respectively. A previous study showed that OFs from type II GO patients have greater proliferative potentials than OFs from type I GO and non-GO patients (52). TGF-β significantly stimulates the proliferation of GO-derived OFs, especially type II OFs, compared to non-GO OFs (52, 53). In our study, CCK8 and EdU assays consistently evidenced an anti-proliferative effect of DHA on GO-derived OFs, which suggests that DHA may be especially beneficial for type II GO patients.

Orbital fibrosis is a vital pathological change in GO. TGF-β1 plays an important role in the pathophysiology of many fibrotic disorders, including GO (41, 54). Treatment of OFs with TGF-β induces expression of fibrotic markers, extracellular matrix production, and differentiation into myofibroblasts (47, 55, 56). Our data showed that DHA remarkably inhibits TGF-β1-induced fibrosis marker expression in GO-derived OFs. This effect was verified by qRT-PCR, immunofluorescence, and western blotting assays that revealed DHA-mediated repression of fibrotic markers such as α-SMA, COL1A1, and CTGF. The mechanisms underlying the antifibrotic effect of DHA were examined by RNA-Seq and western blot analysis, which indicated that DHA effectively inhibited TGF-β1-induced MAPK/ERK and STAT3 signaling in GO-derived OFs. The ERK signaling pathway is a main regulator of cell proliferation, differentiation, adhesion, migration, and survival, and is critically involved in the pathogenesis of various diseases (57, 58). The contribution of dysregulated ERK signaling in hepatic fibrosis, renal fibrosis, and cardiovascular fibrosis is well documented (59–62). A study by Wei et al. in GO-derived OFs suggested that simvastatin and ROCK inhibition exert antifibrotic effects by modulating the ERK signaling pathway (47). Meanwhile, Chen et al. reported that DHA alleviates liver fibrosis and hepatic stellate cell activation in rats by inhibiting PDGF-βR/ERK signaling (63). Here, we provide for the first time evidence that DHA can attenuate OF-mediated fibrosis in GO by suppressing the phosphorylation of ERK induced by TGF-β1.

Activation of STAT3 signaling was also shown to promote fibrosis in pulmonary, cardiac, and liver disease (64–66). Research indicated that DHA can prevent fibrogenesis by inhibiting the activation of STAT3 in *in vitro* and *in vivo* models of liver fibrosis, skin fibrosis, systemic sclerosis, and pulmonary vascular remodeling, among others (67–69). Our study provides also first-time evidence that DHA restrains GO-related fibrosis by inhibiting the phosphorylation of STAT3 in OFs. Indeed, pre-treatment of GO-derived OFs with the ERK1/2 inhibitor U0126 or the STAT3 inhibitor Stattic reproduced the suppression of TGF-β-induced α-SMA expression exerted by DHA. In summary, these results indicated that DHA exerts antifibrotic effects on GO-derived OFs through inactivation of the ERK and STAT3 signaling pathways.

Compared with control OFs, GO-associated OFs are more capable of secreting and responding to inflammatory cytokines, which makes them key players in the amplification of the disease process (70–73). Consistent with previous studies, we found GO OFs were more sensitive to the stimulation of IL-1β than non-GO OFs, characterized by the higher expression of pro-inflammatory factors. DHA was shown to inhibit the secretion of pro-inflammatory cytokines and to exert anti-inflammatory effects in many inflammatory diseases. Wei et al. reported that DHA significantly inhibited the expression of pro-inflammatory cytokines such as IL-6, IL-15, IL-17A, and TNF-α in imiquimod-induced psoriasis-like skin inflammation in mice (74). Meanwhile, Gao et al. reported that DHA alleviated LPS-induced neuroinflammation in mice by reducing IL-1β, IL-6, and TNF-α expression (75). Moreover, the anti-inflammatory effect of DHA has been observed in *in vivo* models of inflammatory bowel disease, rheumatoid arthritis, and systemic lupus erythematosus (31, 76, 77). In the current study, and consistent with previous ones, we found that DHA significantly suppressed IL-1β-induced mRNA levels of pro-inflammatory factors, including IL-6, IL-8, CXCL1, MCP-1, and ICAM-1, in GO-derived OFs and non-GO OFs.

Abnormal accumulation of HA is a core factor leading to orbital tissue expansion and orbital remodeling in GO. Studies showed that IL-1β can stimulate the secretion of HA by GO-derived OFs, and that HA can in turn induce the synthesis of pro-inflammatory mediators in these cells (78–80). Further suggestive of the therapeutic potential of DHA to restrain inflammation and tissue expansion in GO, in this study we found that DHA exposure attenuates IL-1β-induced HA production in GO-derived OFs.

There are several limitations in the present study. First, because of the absence of animal models that mimic the ocular changes affecting patients with GO, the *in vivo* effects and the optimum physiological dose of DHA could not be determined. Second, in this study we focused only on OFs, without considering potential crosstalk between these cells and immunocytes. Despite these limitations, the *in vitro* model used in this study still remains the most widely used strategy to assess OF pathophysiology in GO.

In conclusion, our study is the first to demonstrate the therapeutic effects of DHA in an *in vitro* model of GO. Although further research is clearly warranted, the present data suggest that DHA may represent a cost-effective and safe treatment option for GO.

## Data Availability Statement

The datasets presented in this study can be found in online repositories. The name of the repository and accession number can be found follow: https://www.ncbi.nlm.nih.gov/geo/, accession ID: GSE199688.

## Ethics Statement

The studies involving human participants were reviewed and approved by Ethics Committee of the Zhongshan Ophthalmic Center, Sun Yat-sen University. The patients/participants provided their written informed consent to participate in this study.

## Author Contributions

SY, XW, and WX are jointly responsible for experiment design, experiment implementation, and manuscript writing. HJY are responsible for the guidance of experimental operation and correcting the details of the experimental design. ZX and XS are responsible for collecting sample tissues and conducting primary cell culture. HSY are responsible for the overall control of the experiment and revision of the manuscript. All authors contributed to manuscript revision, read, and approved the submitted version.

## Funding

The material presented in this work was supported with funding from the National Natural Science Foundation (81870689) of China, the Scientific Research Project of Guangdong Provincial Bureau of Traditional Chinese Medicine (20211077) and the Fundamental Research Funds of the State Key Laboratory of Ophthalmology.

## Conflict of Interest

The authors declare that the research was conducted in the absence of any commercial or financial relationships that could be construed as a potential conflict of interest.

## Publisher’s Note

All claims expressed in this article are solely those of the authors and do not necessarily represent those of their affiliated organizations, or those of the publisher, the editors and the reviewers. Any product that may be evaluated in this article, or claim that may be made by its manufacturer, is not guaranteed or endorsed by the publisher.
