# Supplementary material for: Dihydroartemisinin Exerts Antifibrotic and Anti-Inflammatory Effects in Graves’ Ophthalmopathy by Targeting Orbital Fibroblasts
Source: Front Endocrinol (Lausanne). 2022 May 17;13:891922. doi: 10.3389/fendo.2022.891922 (PMC9157422; doi:10.3389/fendo.2022.891922)
Supplement: Supplementary file 1 [file DataSheet_1.docx]

Supplementary Materials

# Supplementary Figure 1


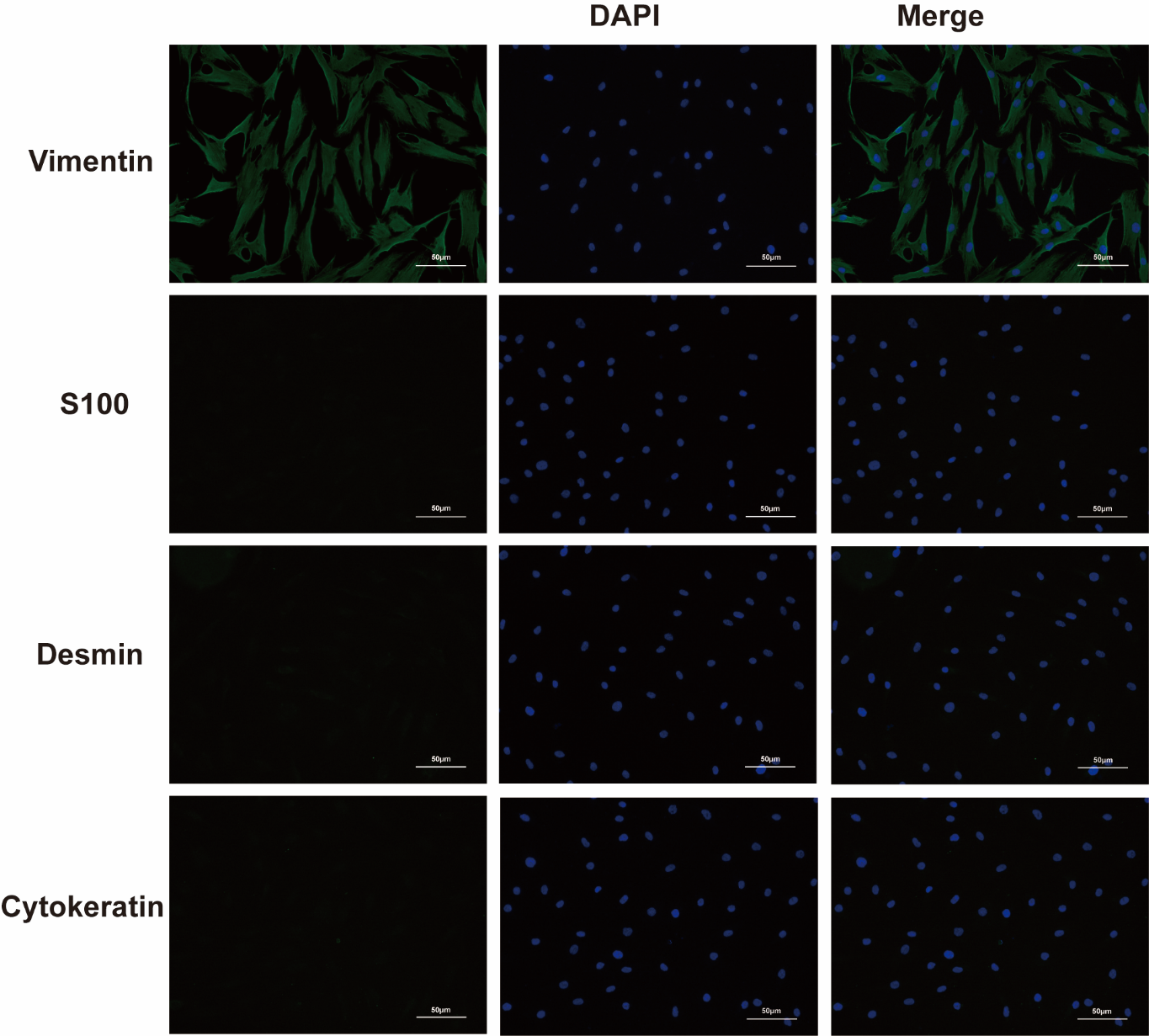


**FIGURE S1.** Immunofluorescence staining for Vimentin, S100, desmin, and cytokeratin in orbital fibroblasts from GO patients (n = 3) and non-GO patients (n = 3). The nuclear counterstain is DAPI (blue). Vimentin was positive expression (green) in the cytoplasm and perinuclear area. Negative expression of S100, Desmin, and cytokeratin was shown above. Scale bar: 50μm.

# Supplementary Figure 2

**
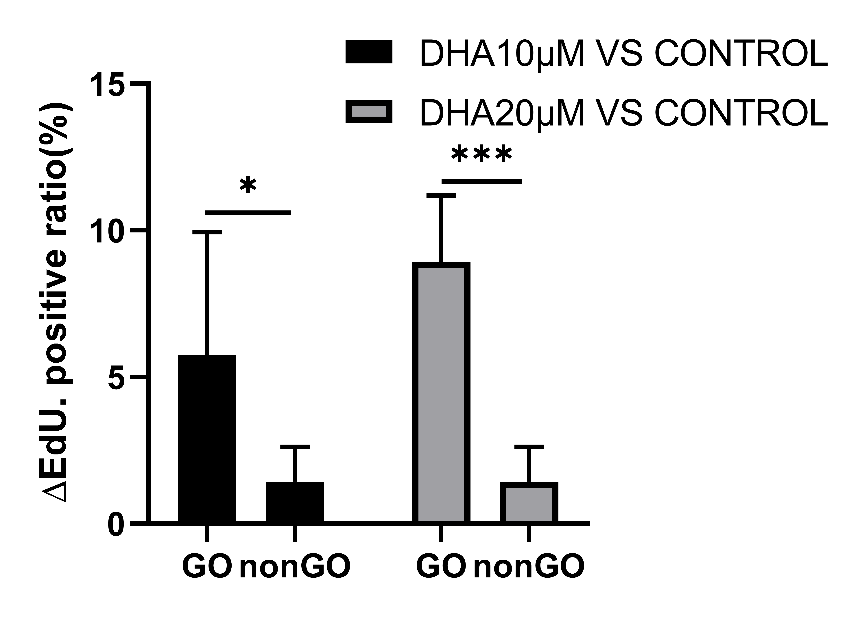
**

**FIGURE S2.** Significant differences between GO OFs and non-GO OFs in ∆EdU. positive ratio.

∆EdU. positive ratio means the difference of EdU. positive ratio between DHA-treated group (10μM, 20μM) and control. *P < 0.05, ~~**P < 0.01,~~ ***P < 0.001~~, **** P < 0.0001 compared with IL-1β alone~~.
